# A Comparative Study on Fat Pattern between Tribal and Non-tribal Girls of Tripura, North-East India

**DOI:** 10.1007/s12098-019-02879-7

**Published:** 2019-02-19

**Authors:** Shilpi Saha, Samir Kumar Sil

**Affiliations:** Department of Human Physiology, Tripura University (A Central University), Suryamaninagar, Tripura 799022 India

**Keywords:** Body composition, Percent of body fat, Fat-free mass, Anthropometry, Tribal, Non-tribal, Tripura

## Abstract

**Objective:**

To examine the body composition including fat patterning among 744 school going Chakma tribal and non- tribal Bengali girls (366 Chakma tribal and 378 Bengali girls), aged 6-12y from North, Unokoti, Dhalai and South District of Tripura.

**Methods:**

The subjects were selected using cluster-random sampling method. The anthropometric measurements of height, weight, triceps and subscapular skinfold were recorded. The body mass index (BMI) was also calculated. The measurements were used to estimate percent body fat (PBF) and fat-free mass (FFM) from skinfolds. Fat mass (FM) and FFM were each divided by height squared to produce the fat-mass index (FMI) and fat-free mass index (FFMI). Body composition was assessed using FM, FFM, FMI and FFMI.

**Results:**

Age-specific mean values of FM ranged from 2.65-6.75 kg (tribal) and 1.92-6.45 kg (non-tribal). Age-specific mean values of FFM ranged from 17.19-29.61 kg for tribals and 15.41-28.44 kg for non-tribals respectively. PBF of tribals was significantly (*p* < 0.01) higher (except 10 y) than non-tribals. FFM and PBF significantly (*p* < 0.01) related with all anthropometric variables.

**Conclusions:**

This study suggested a clear evidence of ethnic variation in fat patterning; Chakma tribal girls showing a greater subcutaneous adiposity in comparison with Bengali girls. These results are important for future investigations in clinical and epidemiological studies to identify the risk of lower or higher adiposity and body composition.

## Introduction

Body composition is a valuable indicator for assessing the adiposity of an individual. Anthropometric measurements are still widely used to assess the body composition in many fields and epidemiological investigations [1]. The amount of body fat differs with age, sex, genetic, environmental and socio-economic conditions and is very useful for assessing the health and nutritional status of a community [2–4]. Body composition status reflects nutritional intakes, losses and needs over time [*i.e.*, fat-free mass (FFM) and FFM index] along with the prevalence of undernutrition. Percent of body fat (PBF) is considered to be a relatively better measure of excess adiposity or obesity. There are several socially deprived communities in India, among which tribal communities are the most vulnerable ones. India has a variety of tribal communities that constitute about 8.6% of the total population [5]; probably the largest tribal community population in the world. A few studies are available on fat mass, fat-free mass and fat-mass index in India [6–12] and from the state of Tripura almost no significant study has been reported. So, the present study has been taken to examine the body composition characteristics including fat distribution among Chakma tribal and non-tribal Bengali girls living in rural areas of Tripura, North-East India.

## Materials and Methods

A cross-sectional study was carried out among 744 school going Chakma tribal and non- tribal Bengali girls (366 Chakma tribal and 378 Bengali girls) aged 6 to 12 y residing in North, Dhalai, Unokoti and South districts of Tripura. Tripura is one of the North- Eastern states of India which is the main homeland of a number of tribes. Geographically, it lies between 22°56′ & 24°32′, North longitude & between 91°10′ & 92°21′, East longitude with a total area of 10,491 Sq.km. According to 2011 census, in Tripura, out of the territories’ total population of 36,71,032, Scheduled tribes numbered 11,66,813, which constitute 31.78% of the total population. The subjects were selected from rural areas (villages) of the state of Tripura, which is the habitat of the Chakma tribal and non-tribal Bengali populations. The school going girls were selected using a stratified multistage clustered random sampling method. Initially 871 girls (Chakma: 423 and Bengali girls: 448) in the age group of 6-12 y were identified and approached to participate in the study. The age of each student was recorded from the school register and their birth certificates. Mothers of the children were included as respondents. Apart from the anthropometric measurements of the children, information on various factors that directly or indirectly affect the nutritional outcome was also obtained. A semi-structured questionnaire was developed which included some baseline information of the parents and the children in regard to nutrition. The questionnaire was finalized by pretest and consultation prior to beginning of the study. All the data were collected after getting the consent from their parents and school authorities. Decimal age calendar is used to determine the student’s decimal age by subtracting the date of birth from the date of data collected. The subjects in various age groups were classified by following the same principle. Children suffering from any systemic disease or those who had undergone any major surgical operation were excluded from the study. Of these 871 girls, 127 of them (Chakma: 42 and Bengali: 85) were excluded from the study as their date of birth were either not valid or they were not in the age group of 6-12 y. This study was conducted in accordance with the ethical guidelines for human experiments, as laid down the Helsinki Declaration of 2000 [13]. The data were collected during the period from August 2015 through April 2016.

Other general information regarding their socio-economic condition, parent’s occupation and education, family income, size, structure and property *etc*., was also recorded. According to the modified Kuppusswamy scale, the socio-economic status of all the children was low [14].

The anthropometric measurements like height, body weight and triceps skinfold thickness (TRSF) and subscapular skinfold thickness (SBSF) of each girl were measured using standard technique [15]. Height was measured by an anthropometer rod (GPM Swiss made) with the head held in the Frankfort horizontal plane and recorded to the nearest 0.1 cm. Weight of the subject, wearing minimum clothing and with bare feet, was taken in the early morning (empty stomach) using a portable weighing machine (Libra) to the nearest 0.5 kg. Body mass index (BMI) was calculated by dividing body weight with standing height (kg/m^2^). Skinfold thickness was measured using the Holtain skinfold caliper with a constant spring pressure of 10 g mm^−2^ on the right side of the body. Mean of the three readings in single location was accepted.

The intra-observer technical error of measurement (TEM) was calculated to determine the accuracy of the measurements by the standard procedure of Ulijaszek and Kerr in 1999 [16]. The TEM was calculated using the following equation:

$$ \mathrm{TEM}=\surd \left({\Sigma \mathrm{D}}^2/2\mathrm{n}\right),\left[\mathrm{D}=\mathrm{difference}\ \mathrm{between}\ \mathrm{the}\ \mathrm{measurements},\mathrm{n}=\mathrm{number}\ \mathrm{of}\ \mathrm{individuals}\right]. $$

Double measurements from the same number of subjects (*n* = 10) had been taken by the same measurer (SS) with six hours of difference. The corresponding calculated values of intra observer showed that, all the measurements were within the normal range of errors reported in literatures [16–18].

Mean and standard error of mean were computed for each anthropometric variable according to the age and ethnicity. Skinfold equation for estimating percentage of body fat (%BF) of Chakma tribal and Bengali girls was used from the method developed by Slaughter et al., in 1988 [19] by using multicomponent model reference measures. This equation uses the sum of triceps and subscapular skinfold thickness (mm) to predict body fat.

$$ \%\mathrm{Body}\ \mathrm{fat}=1.33\left(\mathrm{TRSF}+\mathrm{SBSF}\right)\hbox{--} 0.013{\left(\mathrm{TRSF}+\mathrm{SBSF}\right)}^2\hbox{--} 2.5 $$

$$ \mathrm{Fat}\kern0.35em \mathrm{mass}\ \left(\mathrm{kg}\right)=\mathrm{Body}\kern0.3em \mathrm{weight}\ \left(\mathrm{kg}\right)\ \mathrm{x}\%\mathrm{BF}/100 $$

Fat-free mass (FFM) was calculated by subtracting fat mass (FM) from weight. FM and FFM each were then divided by height-squared to produce the fat-mass index (FMI) and fat-free mass index (FFMI), respectively. Person’s correlation coefficient was used to evaluate the relationship between the anthropometric variables. Student’s t test has been applied to calculate the level of significance. The statistical analysis was performed using statistical package for social science (SPSS) software.

## Results

Sample size for each age group, mean and standard error of mean of height, weight, BMI, SBSF and TRSF of Chakma tribal and non-tribal Bengali girls are presented in Table 1. Height, weight, BMI, SBSF and TRSF (except in 9 y for Chakma tribal girls) between the two populations increased with advances in age. The Chakma girls were observed to be heavier than the Bengali girls. The age specific mean skinfold thickness (*e.g*., TRSF and SBSF) values were observed to be statistically higher among Chakma tribal girls compared to Bengali girls (*p* < 0.01). The age-specific body composition variables like PBF, FM, FFM, FMI and FFMI of two populations are represented in Table 2.

**Table 1:** Age-specific descriptive statistics of height, weight, BMI, Subscapular (SBSF) and triceps (TRSF) skinfold thickness of Chakma tribal and Bengali non-tribal girls of Tripura

Age (years)	N	Height (cm)	Weight (kg)	BMI (kg/ m)^2^	SBSF (mm)	TRSF (mm)
Chakma tribal girls						
6	55	117.58 ± 0.81	19.85 ± 0.31	14.32 ± 0.10	6.04 ± 0.12	7.76 ± 0.14
7	54	122.40 ± 0.77	22.50 ± 0.32	14.87 ± 0.10	6.29 ± 0.13	7.97 ± 0.18
8	51	127.46 ± 0.72	24.38 ± 0.40	14.95 ± 0.13	7.86 ± 0.19	8.22 ± 0.20
9	52	131.33 ± 0.86	26.16 ± 0.44	15.11 ± 0.13	7.17 ± 0.23	7.73 ± 0.21
10	53	133.62 ± 0.69	29.44 ± 0.66	16.40 ± 0.24	7.72 ± 0.29	8.29 ± 0.34
11	51	138.01 ± 0.88	33.57 ± 0.73	17.53 ± 0.23	8.44 ± 0.32	8.77 ± 0.31
12	50	142.95 ± 0.91	36.36 ± 0.77	17.69 ± 0.21	9.87 ± 0.28	9.65 ± 0.21
Bengali non-tribal girls						
6	55	114.66 ± 0.75	17.32 ± 0.29	13.12 ± 0.07	5.14 ± 0.14	6.20 ± 0.17
7	55	119.78 ± 0.72	18.93 ± 0.25	13.17 ± 0.05	5.26 ± 0.11	6.56 ± 0.17
8	54	124.18 ± 0.75	20.85 ± 0.27	13.49 ± 0.05	6.29 ± 0.19	7.35 ± 0.19
9	54	127.76 ± 0.77	22.93 ± 0.35	14.01 ± 0.10	6.49 ± 0.25	7.67 ± 0.24
10	52	131.63 ± 0.69	27.53 ± 0.58	15.82 ± 0.23	7.57 ± 0.22	8.50 ± 0.31
11	55	136.24 ± 0.66	31.14 ± 0.65	16.71 ± 0.25	8.52 ± 0.25	8.64 ± 0.34
12	53	141.75 ± 0.86	34.90 ± 0.82	17.29 ± 0.30	9.84 ± 0.39	9.42 ± 0.44

Values: Mean ± SE; *N* Number of girls

**Table 2:** Age-specific descriptive statistics: mean and standard error (in parenthesis) of anthropometric variables of body composition in Chakma tribal and Bengali non-tribal girls of Tripura

Age (years)	N		PBF (%)		FM (kg)		FFM (kg)		FMI (kg /m)^2^		FFMI (kg /m)^2^	
	Chakma girls	Bengali girls	Chakma girls	Bengali girls	Chakma girls	Bengali girls	Chakma girls	Bengali girls	Chakma girls	Bengali girls	Chakma girls	Bengali girls
6	55	55	13.34 (0.23)	10.84 (0.30)	2.65 (0.07)	1.92 (0.08)	17.19 (0.27)	15.41 (0.21)	1.91 (0.04)	1.43 (0.04)	12.41 (0.09)	11.69 (0.05)
7	54	55	13.77 (0.27)	11.36 (0.26)	3.11 (0.09)	2.17 (0.07)	19.39 (0.26)	16.76 (0.20)	2.05 (0.04)	1.50 (0.04)	12.82 (0.08)	11.67 (0.05)
8	51	54	15.43 (0.33)	13.14 (0.34)	3.78 (0.12)	2.77 (0.10)	20.60 (0.32)	18.07 (0.19)	2.32 (0.06)	1.78 (0.05)	12.64 (0.10)	11.71 (0.05)
9	52	54	14.33 (0.39)	13.58 (0.44)	3.80 (0.15)	3.18 (0.14)	22.35 (0.34)	19.75 (0.23)	2.18 (0.07)	1.91 (0.07)	12.93 (0.10)	12.10 (0.09)
10	53	52	15.21 (0.51)	15.34 (0.46)	4.60 (0.26)	4.34 (0.21)	24.84 (0.45)	23.19 (0.39)	2.53 (0.12)	2.47 (0.10)	13.86 (0.16)	13.35 (0.15)
11	51	55	16.32 (0.48)	16.27 (0.47)	5.57 (0.25)	5.20 (0.25)	28.00 (0.54)	25.94 (0.43)	2.89 (0.11)	2.76 (0.11)	14.64 (0.17)	13.95 (0.17)
12	50	53	18.39 (0.36)	17.90 (0.59)	6.75 (0.23)	6.45 (0.34)	29.61 (0.58)	28.44 (0.52)	3.27 (0.08)	3.16 (0.14)	14.43 (0.16)	14.14 (0.20)

*FFM* Fat-free mass; *FFMI* Fat-free mass index; *FM* Fat mass; *FMI* Fat-mass index; *PBF* Percent body fat

Age specific mean values of FM and FFM were observed to progressively increase with age among both tribal and non-tribal communities. PBF, FMI and FFMI did not exhibit any particular trend between the two communities. The age specific mean value of TRSF was higher at the age of 12 y (9.65 and 9.42 mm) and lowest in 9 y (7.73 mm) and 6 y (6.20 mm) for the Chakma tribal and Bengali girls respectively. The age-specific mean value of SBSF was ranged 6.04 mm to 9.87 mm and 5.14 mm to 9.84 mm among Chakma tribal and Bengali girls, respectively. Age-specific mean BMI values were observed to be significantly (*p* < 0.01) higher among Chakma girls than the Bengalis, especially in the early ages (6-9 y). The age-specific mean BMI values ranged from 14.32 kg/m^2^ to 17.69 kg/m^2^ and 13.12 kg/m^2^ to 17.29 kg/m^2^ among Chakma and Bengali girls, respectively. PBF and FFM values of Chakma girls was significantly (*p* < 0.01) higher than that of Bengali girls. SBSF and TRSF thickness were significantly (*p* < 0.01) higher in Chakma tribal girls than in Bengali girls only between the ages 6-8 y.

**Fig. 1:**
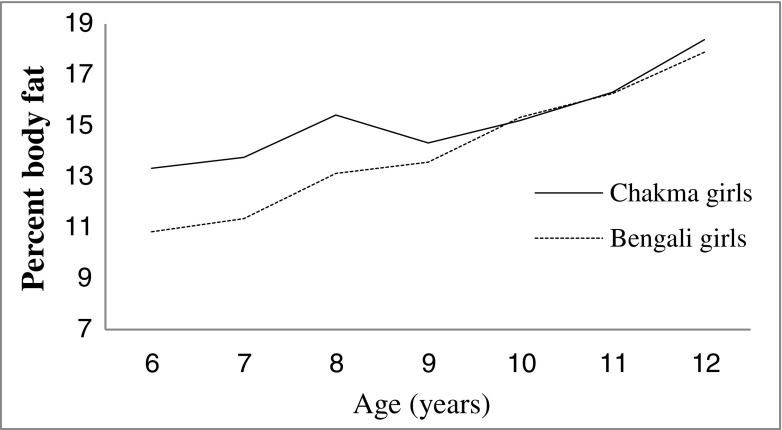
Changes in percent body fat (PBF) of Chakma tribal and Bengali girls

**Fig. 2:**
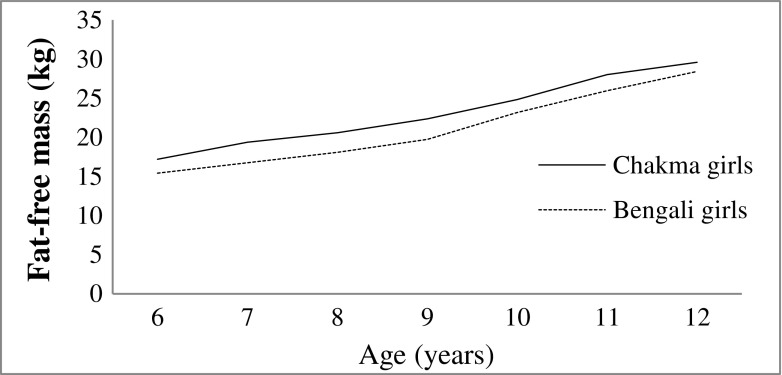
Changes in fat-free mass (FFM) of Chakma tribal and Bengali girls

Figure 1 shows that both the tribal and non-tribal girls gained more PBF between ages between 10 to 12 y, but the Chakma tribal girls gained more fat at early ages (6-9 y). But similar pattern was happening in case of FFM. FFM showed an almost linear positive increment from 6 to 12 y in both the populations (Fig. 2).

**Fig. 3:**
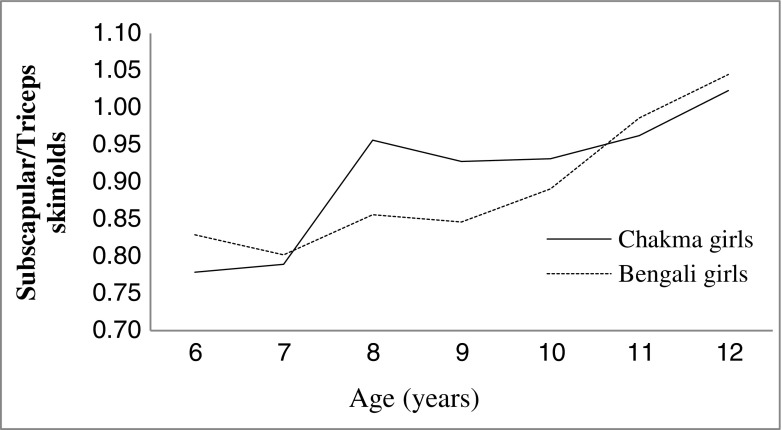
Subscapular to triceps skinfold ratio of Chakma tribal and Bengali girls

Figure 3 shows skinfold ratio of triceps to subscapular between the two populations. Pattern of the subscapular to triceps skinfold ratio, except in ages 8-10 y, was almost same in both the study populations. The ratios for the Chakma tribal and Bengali non-tribal populations showed progressive increment (except in age 9 y) with advancement of age. Chakma tribal girls possessed significantly higher subscapular to triceps ratios than Bengali girls at the age between 8 and 10 y, while the Bengali girls shows slightly higher ratio at the age 6,7,11 and 12 y.

**Fig. 4:**
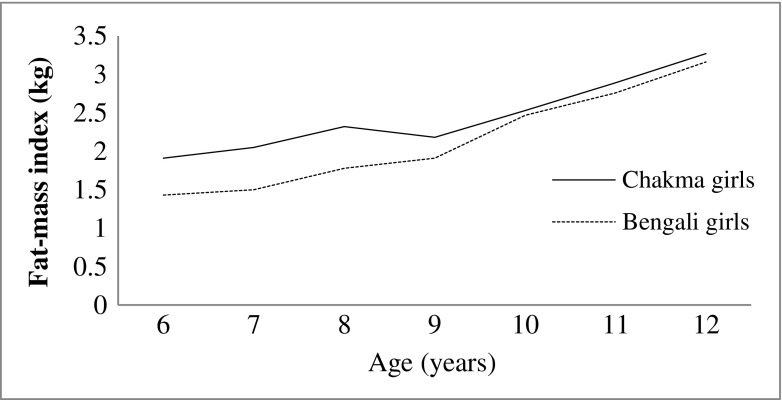
Changes in fat-mass index (FMI) of Chakma tribal and Bengali girls

**Fig. 5:**
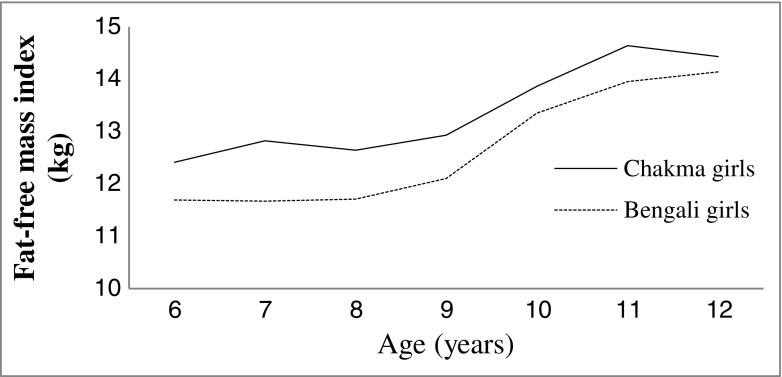
Changes in Fat-free mass index (FFMI) of Chakma tribal and Bengali girls

Graph of FMI showed that body fatness of Chakma girls increases up to the age of 8 y and thereafter a steep fall occurs at the age 9 y and after that it increases up to the age of 12 y. Body fatness of Bengali girls increased with advances in age (Fig. 4). FFMI of Bengali girls showed a steady increase with age, while the Chakma girls showed a different shape of adiposity with a one small dip at the age 12 y. Maximum difference in FFMI between the two populations was found at early adolescence (6-9 y) period (Fig. 5).

Correlation studies show that PBF and FFM were highly correlated (*p* < 0.01) with all anthropometric parameters (Table 3). In both the study populations, the patterns between PBF and anthropometric traits were the same. BMI was also highly correlated with PBF (r = 0.8, r = 0.9; *p* < 0.01) and FFM (r = 0.9; *p* < 0.01) in both the populations.

**Table 3:** Pearson’s correlation between anthropometric measurements of tribal and non-tribal girls of Tripura

	Height	Weight	BMI	SBSF	TRSF	FFM
Chakma tribal girls						
PBF	0.90	0.92	0.86	0.99	0.97	0.91
FFM	0.98	0.99	0.98	0.91	0.87	–
Bengali girls						
PBF	0.99	0.99	0.96	0.87	0.99	0.99
FFM	0.98	0.99	0.98	0.89	0.97	–

All correlation are significant at *p* < 0.01 level

*BMI* Body mass index; *FFM* Fat-free mass; *PBF* Percent body fat; *SBSF* Subscapular skinfold thickness; *TRSF* Triceps skinfold thickness

## Discussion

The distribution and amount of body fat (e.g., FM) and composition of muscle mass (*e.g*., lean body mass or FFM) are important to understand the health outcomes in body composition assessment in infants and children [12, 20–22]. Studies have reported marked ethnic differences in the relationship of visceral and peripheral adiposity [1, 9–11]. But the differences in distribution of fat are evident during early childhood with differences in total body adiposity onset before puberty [23, 24]. Such differences in body fat distribution are mediated by the hormonal fluctuations [25].

Several studies have authenticated different skinfold equations with alternate methods of estimation and recommended the use of the equations of Slaughter et al., in 1988 [19] for the evaluation of body fat among pre-pubertal children [26, 27]. The present study was carried out to evaluate PBF content in order to evaluate the body composition of rural school-going tribal and non-tribal girls of Tripura using this equation of Slaughter et al., 1988 [19]. Furthermore, several studies have assessed body composition characteristics in children utilizing these equations for estimation of PBF among children from both non-Indian [8, 27–29] and Indian ethnic populations [6, 7, 10–12]. The results indicated pronounced ethnic differences in adiposity and body composition measures (*e.g*., PBF, FM, FMI, FFMI) between Chakma tribal and Bengali non-tribal girls (*p* < 0.01) of Tripura. The differences in adiposity measures (PBF, FM and FFM) were also observed to be more prominent with the advancement of age between the two populations.

Present study also suggested a characteristic spurt in the growth of the PBF and FFM. This spurt has been found to coincide with the peak velocities in height and weight [30]. Height is more strongly related to the indicator of lean body mass than to the indicator of adiposity [31]. It is interesting to note that the present study also shows the similar pattern.

An age specific FM value observed in the present study was higher than those obtained from Santal [7], Nepalese [8], Bengalese [1] and Indian [32] girls. The indices of FMI and FFMI therefore suggest a powerful outline for evaluating inter and intra-population variability in body composition and address physique (FFMI) as well as relative adiposity (FMI). The ethnic variation might be attributed to genetic adaptations to ancestral environment and exposure to more existing ecological stresses, as it has been reported that variations in PBF, FM, FMI and FFMI between populations could be due to their ethnic elements [10, 33, 34].

Correlation study suggests that increased PBF and FFM are accompanied by an increase in anthropometric measurements between the two populations. Significant relationship between BMI and PBF, and BMI and FFM, indicate that changes in BMI represent changes in PBF and FFM [35]. Again, the significant correlation between PBF and FFM highlights that the developing pattern of PBF and FFM are similar in tribal and non-tribal populations.

## Conclusions

The present cross-sectional study recommends the evaluation of body composition including fat pattern to improve screening for malnutrition in school children in field and clinical settings in order to reduce chronic malnutrition related morbidity and mortality. The findings of the present study are important for future investigations in the field of epidemiological settings to identify the risk of lower or higher adiposity status and to improve human health through proper intervention programmes.
